# Tools for supporting solution scattering during the COVID-19 pandemic

**DOI:** 10.1107/S160057752100521X

**Published:** 2021-06-25

**Authors:** Lin Yang, Edwin Lazo, James Byrnes, Shirish Chodankar, Stephen Antonelli, Maksim Rakitin

**Affiliations:** a Brookhaven National Laboratory, 745 Brookhaven Avenue, Upton, NY 11973, USA

**Keywords:** mail-in, solution scattering, automation

## Abstract

During the COVID-19 pandemic, synchrotron beamlines were forced to limit user access. Performing routine measurements became a challenge. At the Life Science X-ray Scattering (LiX) beamline, new instrumentation and mail-in protocols have been developed to remove the access barrier to solution scattering measurements. Our efforts took advantage of existing instrumentation and coincided with the larger effort at NSLS-II to support remote measurements. Given the limited staff–user interaction for mail-in measurements, additional software tools have been developed to ensure data quality, to automate the adjustments in data processing, as users would otherwise rely on the experience of the beamline staff, and produce a summary of the initial assessments of the data. This report describes the details of these developments.

## Abstract 2

The development of instrumentation and software to support mail-in solution scattering is presented.

## 1. Introduction   

Recently, we described the instrumentation and software at the LiX beamline of NSLS-II for automated biomolecular solution scattering data collection and processing (Yang *et al.*, 2020 ▸). Mail-in and remote measurements are the logical next steps after a high degree of automation has been achieved, as demonstrated in the macromolecular crystallography community (*e.g.* Robinson *et al.*, 2006 ▸; Okazaki *et al.*, 2008 ▸), and followed by solution scattering (*e.g.* Dyer *et al.*, 2014 ▸). Although making this transition has always been in our plans, the COVID-19 pandemic has made it a necessity. At the time of writing, NSLS-II is controlling personnel density by limiting the number of on-site staff and users, and has instead focused on developing capabilities for remote experiments. Owing to the automation that we already have in place, it has been fairly straightforward for the LiX beamline to transition to all-remote access.

Mail-in access is available to all solution scattering users with approved beam time proposals. As described previously, users who routinely perform solution scattering measurements can obtain recurring access through Block Allocation Groups, whereas those who only need access occasionally can submit rapid access proposals that can be approved for beam time typically a few days after submission. To initiate mail-in measurements, the user first consults the beamline schedule published on the beamline website (https://sites.google.com/view/lixbeamline/) and contacts beamline staff to reserve a spot when they expect the beamline to receive the samples. The user also provides an estimate of the number of samples. The beamline staff then update the schedule once the proposed time of measurement is confirmed. The user can then proceed to preparing samples.

Before shipping the samples, the user submits a safety approval form (SAF) – as required for all experiments, but streamlined for routine solution scattering measurements – with the sample information attached. Samples are tracked based on this user-submitted, container-dependent (see description in Section 2) information to eliminate human error in the process of sample transfer and measurement.

Each day the beamline can measure samples from multiple user groups. The samples are either received in the LiX sample holders (Yang *et al.*, 2020 ▸), or transferred into these sample holders from 96-well plates or individual user containers. Each holder is uniquely identified by a QR code to keep track of sample information. Beamline staff simply need to populate the sample storage unit with all available sample holders, and data collection and processing can proceed automatically. Data directories are shared with users via Globus (https://www.globus.org/) before the measurements, so that they have access to the data and processing results in real time.

In this report, we first describe the sample formats we have chosen for large numbers of mail-in samples, and the instrumentation for transferring and mixing samples. We then describe the software tools for ensuring that the users receive high-quality data even though they do not participate in data collection.

## 2. Sample format   

All beamlines specialized in biomolecular solution typically offer mail-in support in some form. Ideally the samples should be measured from the container in which they are shipped, to reduce the preparation work for the beamline staff and, more importantly, to prevent possible mix-ups of sample identity. The 96-well format is supported by standard liquid-handling equipment and used by many solution scattering beamlines (*e.g.* Round *et al.*, 2015 ▸; Hura *et al.*, 2009 ▸; Nielsen *et al.*, 2012 ▸). The instrumentation at the LiX beamline, however, is not compatible with this format, due to the design choices that we made to optimize the instrument performance (Yang *et al.*, 2020 ▸). We have therefore devised two solutions to reliably track large numbers of samples and reduce the workload of the beamline staff at the same time.

We still have users who have only a handful of samples (<10) for feasibility tests or for in-line SEC measurements. They are free to send their samples in the format of their choosing, although PCR tubes that are compatible with the LiX sample holders are preferred for static measurements. These users are asked to label clearly how each sample should be identified in the data file they receive. The beamline staff then prepare the sample holder and sample measurement spreadsheet as the users would during their on-site visits to the beamline. This format is also suitable for some proprietary users, who require structural characterization on their pre-packaged products.

### 2.1. Shipping box   

**Figure 1:**
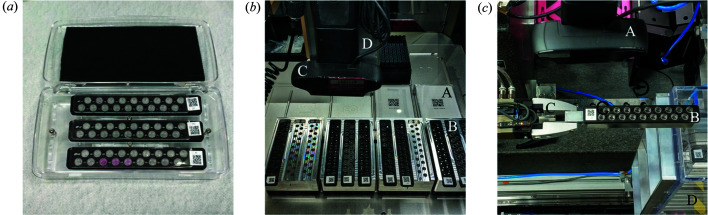
Equipment used in mail-in solution scattering experiments. (*a*) A shipping box with three sample holders received from a user. (*b*) PCR plates (A) and LiX sample holders (B) on the OT2 deck ready for sample transfer. The webcam for QR code reading (C) can be seen in the foreground, mounted on the OT2 pipette arm (D). The QR code labels and lids are still attached to the PCR plates. They will be removed once the labware have been identified and the transfer protocol generated. (*c*) The webcam (A) overlooking a sample holder (B) at the transit position for the Staubli robot (only the gripper is seen, C). Before data collection begins, each sample holder is picked up by the robot to read the QR code, then returned to the storage unit (D).

The samples are measured in 0.2 ml PCR tubes at the LiX beamline. In total, 18 tubes are grouped together in an aluminium sample holder, with a QR code-bearing, 3D-printed cover that secures the tubes in the holder. We designed a sample box that can accommodate up to three LiX sample holders, so that the sample holders can be populated by the users and measured directly once they are received at the beamline. This shipping box is based on an off-the-shelf rectangular container with laser-cut plastic inserts [Fig. 1 ▸(*a*)]. We ship to users PCR tubes with a cover, completed with the QR code and a plug seal that we fabricate from silicone (Sylgard 184, Dow) at the beamline. By sending users PCR tubes, we also bypass the issue that there are size variations between tubes from different vendors and consequent not all tubes fit into our sample holders.

The user provides a measurement spreadsheet that can be directly used for automated measurements, as described previously (Yang *et al.*, 2020 ▸). We require the users to validate the spreadsheet and eliminate errors that would otherwise stop automated data collection using beamline-provided software. The software verifies that the file names conform to the detector software requirements, that there are no duplicates within the same sample holder and that each sample is assigned a buffer for background subtraction. By default, a new buffer measurement is required for every five samples, to account for possible drift of empty-cell scattering. Finally, the validation also embeds user information (proposal and SAF numbers) into the spreadsheet for use during automated data collection. The user assigns each sample holder a name no longer than four letters long. During the process of validation, a universally unique identifier (UUID) is generated for each holder. Once the beamline receives this measurement spreadsheet as an attachment to the SAF via the NSLS-II proposal system, the beamline staff will print the QR codes, completed with the sample holder identifier (hence the length limitation), using an EPSON LW-PX800 label printer, directly from the spreadsheet. The identifier helps the user to differentiate between the sample holders in the shipping box without having to scan the QR code. An example measurement spreadsheet and validating *Jupyter* notebook are given in the supporting information.

### 2.2. 96-well plate   

The 96-well format offers a greater capacity for samples and additional flexibility in sample preparation. To transfer the samples to the LiX sample holders, we have commissioned an Opentrons (https://opentrons.com/) OT2 liquid-handling robot. Along with each plate, the user provides a sample information spreadsheet (different from the measurement spreadsheet for samples in shipping boxes) to identify each sample and the associated buffer. Only 9 of the 12 wells in each row are used for samples, matching the two 9-position rows in each LiX sample holder, therefore making it easier to automatically generate the transfer protocol. At the same time, the three additional wells can be used for buffer or other chemicals for the purpose of creating concentration or mixing series. An example spreadsheet is included in the supporting information.

The sample information spreadsheet is also validated for sample name validity and consistency. Each sample is identified by its volume, or the transfer volumes and the source locations if mixing is required. During validation the software produces a plate identification label in the form of a web page (see the supporting information for an example), consisting of the proposal number, the SAF number and a two-digit plate identifier, together with a QR code that encodes the same information. The user prints the label and attaches it to the plate for shipment. Once the beamline receives the plate and information spreadsheet, a transfer protocol is generated automatically by software, together with a measurement spreadsheet that will be used for data collection. The protocol is then executed on the OT2 robot to transfer the samples from the plate to the LiX sample holders [Fig. 1 ▸(*b*)]. The details are discussed in the next section.

## 3. OT2 integration   

The OT2 robot is based on a Raspberry Pi single-board computer. Opentrons provides an app for executing transfer protocols. OT2 also runs a *Jupyter* hub for developing Python codes that can run as stand-alone scripts. The app is available for all major computer platforms and connects to the robot via either WiFi or USB. In order to access other data on the beamline network, we employ a Beaglebone, another type of single-board computer that we have used extensively for instrumentation control, to connect to the Raspberry Pi via USB and to the beamline network via Ethernet. By using secure shell tunneling, the beamline staff can control the robot using the app from the beamline workstations. From there, beamline staff can also run the software for generating transfer protocols and measurement spreadsheets from user-supplied sample information spreadsheets, which are downloaded ahead of the measurements from the proposal system into the data directory shared with the user.

The OT2 manufacturer provides a library of standard labware that can be used in transfer protocols and allows custom labware to be defined. To minimize delay, for sample shipping we ask users to use the Bio-Rad 0.2 ml 96-well PCR plate, which is included in the OT2 library, or labware with identical dimensions. By using the same labware for all transfers, we are also able to fine-tune parameters such as the distance from the end of the pipette tip to the bottom of the well during sample transfer to minimize the amount of unused samples. To receive sample transfers, we group three sample holders together into a labware, which physically corresponds to an adaptor that occupies the footprint of a 96-well plate [Fig. 1 ▸(*b*)]. The OT2 can accommodate up to 12 standard-plate-size labwares on its deck. Taking into account the tips needed for sample transfer, the OT2 can therefore operate on up to two full plates and 12 LiX sample holders in each transfer run.

To transfer samples from plates to LiX holders, each plate and holder must be identified. During the validation of the sample information spreadsheet, the software reports the numbers of sample holders and tip racks needed for the transfer. Beamline staff first centrifuge the sample plates and load them into the OT2, together with the required number of sample holders, each affixed with a QR code corresponding to a randomly generated UUID, again printed using the EPSON printer. In contrast to the sample holders in the shipping box, whose UUID-encoded QR codes are correlated with the user-specified sample holder identifier, beamline staff do not need to be aware of the identity of the sample holders transferred from plates. Instead, this correlation is tracked by software and recorded in the automatically generated measurement spreadsheet, which is then recalled during data collection.

Once all sample plates and sample holders are loaded onto the OT2 deck, the beamline staff run a Python function from the beamline *Jupyter* hub to remotely activate a Python script on the OT2 Raspberry Pi to scan each deck location to read and report the QR code (see details in Section 5) on each sample holder or plate. Based on the identity of the labware and the sample information spreadsheet, this function produces a protocol file for sample transfer and mixing, as well as the measurement spreadsheet for data collection, completed with the user information (proposal and SAF numbers) from the sample information spreadsheets.

## 4. Data collection   

Using the process described above, by the time of measurement all sample holders will have a unique identifier encoded in its QR code, which in turn is correlated to a measurement scheduling spreadsheet, with the user information already embedded. All available sample holders are centrifuged, regardless of how they were received, and populated into the storage unit, which has a capacity of up to 20 sample holders (360 samples) (Yang *et al.*, 2020 ▸). Ideally, automated measurements can be started twice a day, in the morning for the daytime measurements, and at the end of the day for the overnight measurements.

To initiate automated data collection, the Staubli robot (Yang *et al.*, 2020 ▸; Lazo *et al.*, 2021 ▸) picks up each sample holder from the storage unit so that the QR code can be read. The software verifies that the sample information is available for all holders before proceeding to start measurements based on a Python dictionary compiled from the sample holder information, grouped by owner of the samples. For each owner, a data directory is created if it does not yet exist. The link to the data directory is shared with the user via Globus, which is typically done manually and ahead of automated data collection, since it currently requires interactive authentication. As data collection proceeds, data files are packaged for each sample holder and processed as previously described (Yang *et al.*, 2020 ▸).

## 5. QR code decoding   

We rely on QR code decoding to identify the plates and sample holders. Initially, we tested a USB-based barcode reader (Unitech FC75) in the so-called presentation mode. The reader is triggered automatically whenever it recognizes symbols that can be decoded. Unfortunately, we soon discovered that it cannot always trigger reliably when a QR code is presented. In addition, the proximity of the three QR codes in the LiX sample holder labware further confuses the device, when multiple QR codes are within its field of view. This is especially problematic if not all three sample holders are present. The QR code reader is expected to report that no code can be read if the sample holder is absent. In practice, however, the reader returns the QR code from an adjacent location, resulting in misidentification of sample holders. We therefore turned to USB webcams, due to their small sizes and that they can be read directly from the BeagleBone connected to the OT2 robot, and use software to decode QR codes from specific parts of the image.

The current device we use is the Logitech C930e. The selection of the image size, as well as controls for zoom, focus and exposure time, can all be accessed using standard Linux software v4l2-ctl. The webcam for reading QR codes in the OT2 is mounted on the pipette arm [Fig. 1 ▸(*b*)], which moves only in the plane parallel to the deck. The webcam for reading QR codes during data collection is mounted just above a transit position in the Staubli robot trajectory, after the robot picks up a sample holder from the sample storage unit [Fig. 1 ▸(*c*)]. In either case the webcam is located at ∼20–30 cm from the object.

**Figure 2:**
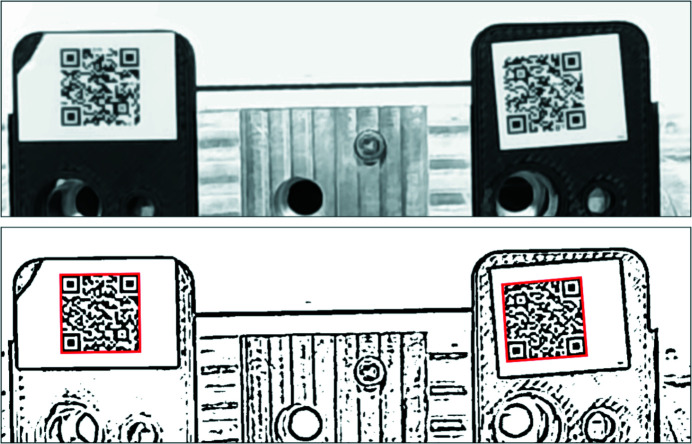
An example of QR code decoding using the webcam. The top image is as captured using *OpenCV*. The focus of the camera has been adjusted with the best effort. The bottom image is after preprocessing using Gaussian adaptive thresholding. Even though the original image is probably over exposed and not in sharp focus, the two QR codes are successfully decoded. The image size is 1280 × 400 pixels. The red boxes in the bottom image are the polygons returned by pyzbar.

We use pyzbar (https://github.com/NaturalHistoryMuseum/pyzbar/) to read the QR codes from the images. The *decode*() function call returns the successfully decoded QR codes as well as their locations. Therefore, in the case of the LiX sample holder labware in OT2, which can contain up to three QR codes, only one image needs to be taken. We can then determine which sample holder each QR code belongs to based on its location. However, the raw images from the webcam typically cannot be decoded (Fig. 2 ▸). We therefore utilize adaptive Gaussian thresholding under *OpenCV* (https://opencv.org/) to preprocess the images. This function, *adaptiveThreshold*(), requires two arguments: block size and background. We found that the same values may not work for all images, or even all QR codes in the same image. To ensure successful decoding of all QR codes, we loop over a range of values for each argument, until all expected QR codes (three for the sample holder labware in OT2, one for plates or sample holders in the storage unit) are decoded, or the end of the loop has been reached. In the latter case, *i.e.* when either fewer than three sample holders are present in the labware or one of the sample holders does not have a QR code, the software simply reports the codes that have been found. Lighting seems critical for successful decoding. We found glare from surrounding light sources to be particularly detrimental. We adjust the camera exposure time based on its specific environment (either sample storage or OT2). The Python code from reading the camera is set up as a server that listens for read requests from a network socket and returns decoded UUIDs. This code runs on the BeagleBone for the OT2 webcam. For the sample storage webcam, it runs on a nearby x86-based EPICS IOC server.

## 6. Other software tools   

In addition to supporting sample preparation and data collection as described above, we also developed software tools for data processing and analysis to support mail-in measurements. These tools are collected under the *py4xs* and *lixtools* packages, available on Github (https://github.com/NSLS-II-LIX) and PyPI (https://pypi.org/). The functions described above can be found in the *mailin*, *ot2* and *webcam* modules of the *lixtools* package.

### 6.1. Beam center verification   

As a data quality control measure, each day we collect standard scattering patterns from silver behenate, in addition to empty cell and water scattering, to ensure that the correct detector configurations are used for data processing. Since the configurations are not expected to change significantly, the non-interactive command line tool *pyFAI-recalibrate* (Kieffer *et al.*, 2020 ▸) has been used to refine the beam center position. This tool is now obsolete in the most recent version of *pyFAI*. A more interactive function therefore has been implemented to take inputs from the PONI files saved by *pyFAI-calib2*. From time to time, we also take horizontal and vertical line cuts through the beam center position from the very low angle part of the SAXS data (from any sample), and compare the scattering intensity on both sides of the beam. If the beam center is correct, the two sides should be identical.

Comparing two scattering profiles and assessing the similarity between them could be achieved using generic numerical tools such as cross correlation. For solution scattering, *cormap* (Franke *et al.*, 2015 ▸) is a popular new tool. The basic idea behind this method is as follows: when comparing two sets of scattering intensity that are considered identical, statistical noise dictates that there are equal chances to observe a higher value in either dataset at a given scattering angle (or the value of the scattering vector, *q*). The result therefore can be described using the same statistics for a series of coin tosses. The longest consecutive appearance of greater values in one dataset than the other, or the longest run of heads in a coin toss series, is predictable (Schilling, 1990 ▸) and can be used to assess dataset similarity. This method was discussed by Franke *et al.* and has also been used for evaluating radiation damage in solution scattering measurements (Brooks-Bartlett *et al.*, 2017 ▸).

**Figure 3:**
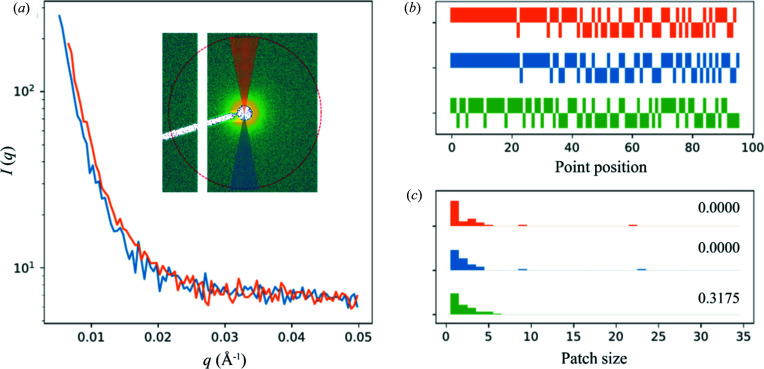
Beam center verification based on the assumption that solution scattering intensity is expected to be centro-symmetric. (*a*) Scattering intensity on either side of the beam along a vertical ‘line cut’ is shown, created by taking the azimuthal average within two pie slices above and below the beam position in the 2D scattering pattern, as shown in the inset, where the red circle indicates *q* = 0.05 Å^−1^. A horizontal cut is also examined in practice. (*b*) The bar charts show the ‘coin toss’ comparison between the two sets of data, for the current beam center position (blue), as well as after the center position is adjusted by 1 data point in either direction (green and orange). (*c*) The comparison is also plotted as histograms of the patch size (consecutive appearance of 1 or −1 in the bar charts). *P*-values for each case are shown. The null hypothesis is that the ‘coin toss’ result is random, and therefore the two datasets are considered statistically identical. In this particular case, the beam center position may need to be adjusted.

The correlation map as described in the references above is presented as a 2D map, which obscures the rationale behind this method. And the significance of the off-diagonal elements of the map is also not clear, in the context of similarity evaluation between datasets. Instead, we plot the comparison of two datasets simply as a bar chart of heads versus tails (1 for one dataset having higher intensity than the other, and −1 otherwise), together with a histogram of the consecutive appearance of 1 or −1, or the patch size as discussed by Franke *et al.* For beam center verification, long stretches of heads or tails suggest that the beam center position is off. The same plot is repeated after the beam center position is adjusted by one data point (in *q*) in either directions, from the currently assumed center (Fig. 3 ▸). If the beam center is indeed off, an improvement would be observed after shifting the center in one of the directions. This is implemented as a method of the *h5xs* class under *py4xs.hdf* and is used to help decide whether a new silver behenate pattern needs to be recorded and the detector configuration updated.

### 6.2. Automatic scaling factor estimate   

As discussed previously in detail (Yang *et al.*, 2020 ▸), at the LiX beamline, scaling and subtraction of buffer scattering is based on the magnitude of the water peak at *q* ≃ 2 Å^−1^. However, since the water peak overlaps with the scattering from proteins, after the water peak magnitudes from the sample and the buffer are matched, the buffer scattering intensity must be manually scaled down (by a factor of ∼0.995) to account for the non-zero scattering intensity contribution from proteins and avoid artifacts in the buffer-subtracted data.

**Figure 4:**
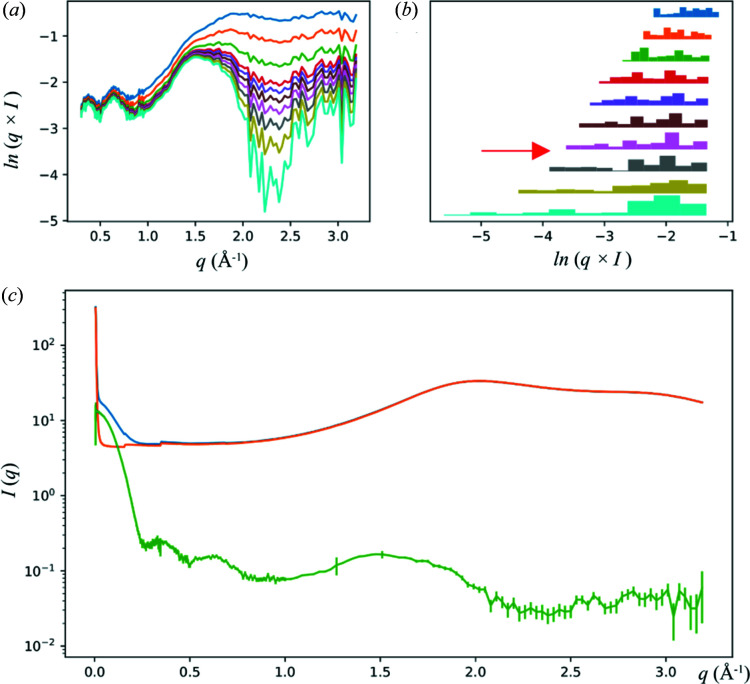
Automated determination of the buffer subtraction scaling factor. The scattering data from a lysozyme sample are shown as an example. The progression of the scaling factor adjustment is shown as (*a*) weighted scattering intensity *q* × *I* (only data at *q* > 0.3 Å^−1^ are used) and (*b*) the span between the maximum and minimum for the buffer-subtracted data. The data in (*b*) are presented as a series of histograms. The color code is the same for (*a*) and (*b*). From top to bottom, the corresponding scaling factors used for buffer subtraction are 0.990, 0.993, 0.995, 0.996, 0.9963, 0.9966, 0.9969, 0.9972, 0.9975, 0.9978. The final subtraction result is shown in green in (*c*), together with the protein solution scattering (blue) and scaled buffer scattering (orange), using a scaling factor of 0.9970. The final scaling factor falls between two plotted values, as indicated by the red arrow in (*b*).

Clearly this is not practical for automated data collection. Therefore we have devised a method, implemented in the *estimate_scaling_factor*() function under *py4xs.slnxs*, to estimate the scaling factor based on two simple criteria: that the scattering intensity must be non-negative and that it also should not vary drastically beyond *q* = 0.3 Å^−1^, since there simply cannot be any corresponding structural features. In practice, we require the subtracted intensity not to have a minimum under the water peak, which would suggest over subtraction. However, as is the case for all atomic scattering factors, the scattering intensity does drop off slightly with increasing *q*. Therefore, the minimum is searched in the subtracted data after multiplication by *q*. Furthermore, we require that the increase in the dynamic range of the data, as measured by the span between the highest and lowest intensities on a logarithmic scale, not to exceed a threshold as a consequence of adjusting the scaling factor, which is another outcome that can be expected of over-subtraction of buffer scattering. The scaling factor is optimized iteratively. It is increased gradually from 0.9. The iteration stops once one of the above criteria is violated. The step size for the scaling factor adjustment, which is initially set to 0.01, is then decreased by a factor of 10. The process is repeated until the desired precision for the scaling factor is achieved (typically 0.0001). Though these criteria are purely empirical, they seem to work well in practice (Fig. 4 ▸). Of course the user can still adjust this scaling factor manually if they so choose, using the data processing graphical user interface (GUI) (Yang *et al.*, 2020 ▸).

### 6.3. Data processing summary report   

A *Jupyter* notebook-based GUI allows users to browse data in the hdf5 file and conduct initial assessment of the data (Yang *et al.*, 2020 ▸). The drawback of this GUI, or any graphical interface in general, is that it requires interactive inputs, which are not practical for large amounts of data. We therefore provide users with a data processing summary report for each sample holder, so that they can have a quick glance of all the data. This report is generated using *nbconvert* from a template *Jupyter* notebook based on the same functions used in the data processing GUI. All Python codes are removed so that the users can focus on the data and the assessment using tools available under *ATSAS* (Franke *et al.*, 2017 ▸). The interface to *ATSAS* is accomplished by processing the data saved in temporary files and interpreting the command line output from *ATSAS*, implemented under *lixtools.atsas*. An example report can be found in the supporting information.

### 6.4. Modeling pipeline   

**Figure 5:**
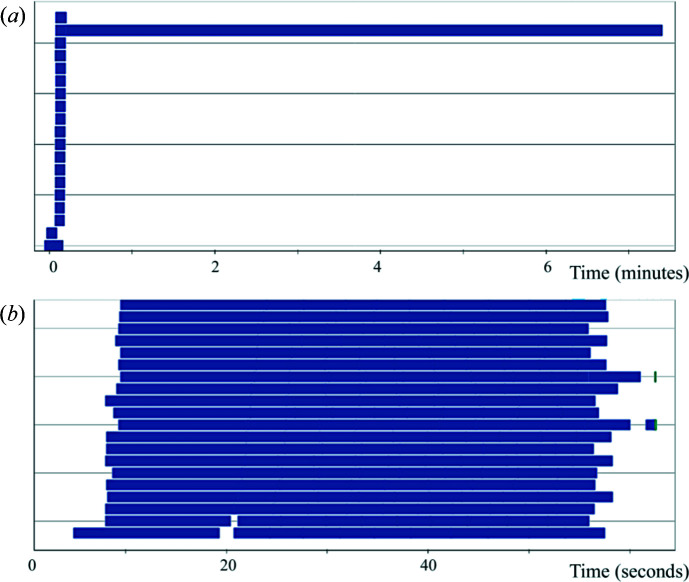
Dask dashboard task stream charts for two modeling runs using *DAMMIF*. Each bar in the chart represents a task that is executed by a worker process. The length of the bar is the execution time. In total, 20 work processes are used for each run. Both runs generated 20 bead models in fast mode, based on the data from a lysozyme sample. *DAMAVER* with the ‘-a’ option is then used to merge these models in (*a*). In contrast, in (*b*) the *SUPCOMB* tasks are distributed to the worker processes and performed in parallel. Note the difference in the time axis. The saving in total run time due to parallelization is obvious. While each *DAMMIF* model only took ∼15 s to complete, the single-threaded *DAMAVER* [the longest bar in (*a*)] lasted ∼7 min.

Once the data quality is judged to be satisfactory, the user has the option to send the data to a modeling pipeline we implemented using *Dask* (https://dask.org/), a Python library for distributed computing. The pipeline is accessible using the *model_data*() function under *lixtools.modeling*. The benefit of using *Dask* is that the exact same code can run on different computing resources, as long as the *Dask* client is defined appropriately. At the LiX beamline the pipeline runs on the NSLS-II cluster using Slurm. The same pipeline can also run on a laptop, using thread-based parallelization. By default, the pipeline produces 20 models, using *DAMMIF* and a pool of worker processes through *Dask*. The resulting models are then aligned and merged. Although this could be done using *DAMAVER* under *ATSAS* with the ‘-a’ option, a single-threaded run takes a long time to complete. We therefore break down this task into smaller SUPCOMB runs [a total of *N*(*N* − 1)/2, where *N* is the number of models], again distributed through *Dask* to the cluster. This reduces the total run time significantly. The aligned models are then selected and averaged together. In addition, *Dask* allows each process to start as soon as the individual models it depends on become available. The entire process is shown graphically in Fig. 5 ▸, as seen from the *Dask* dashboard. It is clear that computation was performed in parallel by all 20 worker processes with minimal delay other than waiting for the work processes to become available on the shared cluster.

This pipeline can also use *DENSS* (Grant, 2018 ▸) to generate and align models. This can be particularly helpful for data that extend to high *q*, which is always the case at LiX. The overall data flow in the pipeline is identical for *ATSAS*/*DAMMIF* and *DENSS*, with the exception of a couple of significant differences. First, DENSS generates electron density maps instead of bead models. Therefore alignment and merging require the tools included in the *DENSS* package. Second, *ATSAS* measures model similarity by the normalized spatial discrepancy (NSD), whereas *DENSS* uses a correlation score. For a perfect match, NSD = 0 while the correlation score is 1. In practice, we use a value of 1 minus the correlation score. The same code then can be reused for both *ATSAS* and *DENSS*.

## 7. Summary   

We have described the instrumentation and software at the LiX beamline for supporting mail-in solution scattering measurements. We find this mode of beamline operation helpful in providing more flexibility to both users and beamline staff. More capacity for measurements is realized by virtue of unattended measurements, which can be set up for a large number of samples from multiple groups of users. We hope this will encourage users to send more samples to the beamline, by treating the beamline as an extension of the repertoire of their lab instrumentation. On the other hand, we are also keenly aware that sample quality may not always be assured. Accordingly, we are promoting the awareness of these issues during the LiX solution scattering workbench, a once-per-cycle user training class now running virtually. Even high-quality data may be neglected after the measurements, simply because it is very easy to obtain a lot of data. We therefore have staff follow up with users using the summary report described above. Given the success so far, we anticipate that most users will continue to use mail-in even after the restrictions due to the COVID pandemic are lifted.

## Supplementary Material

Example sample list spreadsheet for samples in a 96-well plate. DOI: 10.1107/S160057752100521X/ye5004sup1.xlsx

Example measurement spreadsheet for samples already loaded in 18-position sample holders. DOI: 10.1107/S160057752100521X/ye5004sup2.xlsx

Jupyter notebook demonstrating the validation of spreadsheets. DOI: 10.1107/S160057752100521X/ye5004sup3.txt

Example label for a 96-well plate. DOI: 10.1107/S160057752100521X/ye5004sup4.html

Example data processing report. DOI: 10.1107/S160057752100521X/ye5004sup5.html
