# Supplementary material for: Tools for supporting solution scattering during the COVID-19 pandemic
Source: J Synchrotron Radiat. 2021 Jun 25;28(Pt 4):1237–44. doi: 10.1107/S160057752100521X (PMC8284406; doi:10.1107/S160057752100521X)
Supplement: Supplementary file 4 [file s-28-01237-sup4.html]

332405-356113-01
