# Supplementary material for: Tools for supporting solution scattering during the COVID-19 pandemic
Source: J Synchrotron Radiat. 2021 Jun 25;28(Pt 4):1237–44. doi: 10.1107/S160057752100521X (PMC8284406; doi:10.1107/S160057752100521X)
Supplement: Supplementary file 5 [file s-28-01237-sup5.html]

Yogesh\_1\_report


### NSsp13\_4

```
Gunier fit: quality = 95.0 %, I0 = 15.60 +/- 0.03 , Rg = 29.20 +/- 0.28
GNOM fit: quality = 0.96, Dmax = 95.60, Rg = 29.36
Volume estimate: 81364.8 (datporod), 76022.6 (MoW)
MW estimate: 62.7 kDa (MoW)
```

### Nsp13\_5

```
Gunier fit: quality = 95.0 %, I0 = 7.98 +/- 0.03 , Rg = 28.70 +/- 0.27
GNOM fit: quality = 0.94, Dmax = 93.96, Rg = 29.00
Volume estimate: 66963.3 (datporod), 64309.9 (MoW)
MW estimate: 53.1 kDa (MoW)
```

### Nsp16-10\_6

```
Gunier fit: quality = 94.0 %, I0 = 57.70 +/- 0.19 , Rg = 45.70 +/- 1.50
GNOM fit: quality = 0.71, Dmax = 190.28, Rg = 48.99
Volume estimate: 140760.0 (datporod), 120435.0 (MoW)
MW estimate: 99.4 kDa (MoW)
```

### Nsp16-10\_7

```
Gunier fit: quality = 83.0 %, I0 = 19.30 +/- 0.09 , Rg = 33.00 +/- 3.20
GNOM fit: quality = 0.76, Dmax = 115.06, Rg = 34.09
Volume estimate: 85517.5 (datporod), 75781.5 (MoW)
MW estimate: 62.5 kDa (MoW)
```

### Nsp16-10\_8

```
Gunier fit: quality = 92.0 %, I0 = 4.25 +/- 0.03 , Rg = 26.40 +/- 0.77
GNOM fit: quality = 0.90, Dmax = 79.20, Rg = 26.27
Volume estimate: 40458.0 (datporod), 43048.5 (MoW)
MW estimate: 35.5 kDa (MoW)
```

### RdRP\_1

```
Gunier fit: quality = 93.0 %, I0 = 349.00 +/- 3.10 , Rg = 98.40 +/- 12.00
GNOM fit: quality = 0.69, Dmax = 445.57, Rg = 109.00
Volume estimate: 759251.0 (datporod), 417993.0 (MoW)
MW estimate: 344.9 kDa (MoW)
```

### RdRp\_2

```
Gunier fit: quality = 89.0 %, I0 = 75.80 +/- 0.15 , Rg = 43.50 +/- 2.00
GNOM fit: quality = 0.79, Dmax = 160.56, Rg = 42.60
Volume estimate: 172729.0 (datporod), 139903.0 (MoW)
MW estimate: 115.4 kDa (MoW)
```

### RdRp\_3

```
Gunier fit: quality = 79.0 %, I0 = 21.30 +/- 0.07 , Rg = 42.20 +/- 15.00
GNOM fit: quality = 0.74, Dmax = 130.87, Rg = 38.72
Volume estimate: 141123.0 (datporod), 117509.0 (MoW)
MW estimate: 96.9 kDa (MoW)
```

### RdRp\_Nsp13\_nsp16-10\_AIDP\_14

```
Gunier fit: quality = 76.0 %, I0 = 74.90 +/- 0.34 , Rg = 44.80 +/- 19.00
GNOM fit: quality = 0.77, Dmax = 167.81, Rg = 46.12
Volume estimate: 175149.0 (datporod), 144644.0 (MoW)
MW estimate: 119.3 kDa (MoW)
```

### RdRp\_Nsp13\_nsp16-10\_AIDP\_15

```
Gunier fit: quality = 79.0 %, I0 = 203.00 +/- 2.10 , Rg = 55.10 +/- 21.00
GNOM fit: quality = 0.72, Dmax = 238.93, Rg = 59.87
Volume estimate: 284094.0 (datporod), 207582.0 (MoW)
MW estimate: 171.3 kDa (MoW)
```

### RdRp\_Nsp16-10\_11

```
Gunier fit: quality = 82.0 %, I0 = 267.00 +/- 0.56 , Rg = 51.00 +/- 1.70
GNOM fit: quality = 0.79, Dmax = 194.90, Rg = 51.72
Volume estimate: 245845.0 (datporod), 195096.0 (MoW)
MW estimate: 161.0 kDa (MoW)
```

### RdRp\_Nsp16-10\_12

```
Gunier fit: quality = 83.0 %, I0 = 253.00 +/- 0.93 , Rg = 98.70 +/- 18.00
GNOM fit: quality = 0.69, Dmax = 393.82, Rg = 96.48
Volume estimate: 627838.0 (datporod), 385409.0 (MoW)
MW estimate: 318.0 kDa (MoW)
```

### RdRp\_Nsp16-10\_13

```
Gunier fit: quality = 86.0 %, I0 = 29.20 +/- 0.10 , Rg = 41.30 +/- 2.20
GNOM fit: quality = 0.78, Dmax = 157.83, Rg = 42.13
Volume estimate: 149633.0 (datporod), 122940.0 (MoW)
MW estimate: 101.4 kDa (MoW)
```

### RdRp\_nsp13\_10

```
Gunier fit: quality = 77.0 %, I0 = 50.90 +/- 0.25 , Rg = 44.20 +/- 16.00
GNOM fit: quality = 0.80, Dmax = 153.64, Rg = 43.68
Volume estimate: 163938.0 (datporod), 133774.0 (MoW)
MW estimate: 110.4 kDa (MoW)
```

### RdRp\_nsp13\_9

```
Gunier fit: quality = 72.0 %, I0 = 136.00 +/- 1.50 , Rg = 54.70 +/- 20.00
GNOM fit: quality = 0.72, Dmax = 230.86, Rg = 59.37
Volume estimate: 263596.0 (datporod), 195968.0 (MoW)
MW estimate: 161.7 kDa (MoW)
```
